# Plasmolysis: Loss of Turgor and Beyond

**DOI:** 10.3390/plants3040583

**Published:** 2014-11-26

**Authors:** Ingeborg Lang, Stefan Sassmann, Brigitte Schmidt, George Komis

**Affiliations:** 1 Cell Imaging and Ultrastructure Research, University of Vienna, Althanstrasse 14, A-1090 Vienna, Austria; E-Mails: stefan.sassmann@univie.ac.at (S.S.); brigitte.schmidt@univie.ac.at (B.S.); 2 CR-Hana, Palacký University Olomouc, Šlechtitelů 586/11, 783 71 Olomouc-Holice, Czech Republic; E-Mail: georgios.komis@upol.cz

**Keywords:** *Arabidopsis* hypocotyl, cytoskeleton, microtubules, actin microfilaments, plasmolysis, deplasmolysis, GFP-MAP4, GFP-TUA6, GFP-ABD

## Abstract

Plasmolysis is a typical response of plant cells exposed to hyperosmotic stress. The loss of turgor causes the violent detachment of the living protoplast from the cell wall. The plasmolytic process is mainly driven by the vacuole. Plasmolysis is reversible (deplasmolysis) and characteristic to living plant cells. Obviously, dramatic structural changes are required to fulfill a plasmolytic cycle. In the present paper, the fate of cortical microtubules and actin microfilaments is documented throughout a plasmolytic cycle in living cells of green fluorescent protein (GFP) tagged *Arabidopsis* lines. While the microtubules became wavy and highly bundled during plasmolysis, cortical filamentous actin remained in close vicinity to the plasma membrane lining the sites of concave plasmolysis and adjusting readily to the diminished size of the protoplast. During deplasmolysis, cortical microtubule re-organization progressed slowly and required up to 24 h to complete the restoration of the original pre-plasmolytic pattern. Actin microfilaments, again, recovered faster and organelle movement remained intact throughout the whole process. In summary, the hydrostatic skeleton resulting from the osmotic state of the plant vacuole “overrules” the stabilization by cortical cytoskeletal elements.

## 1. Introduction

**Figure 1:**
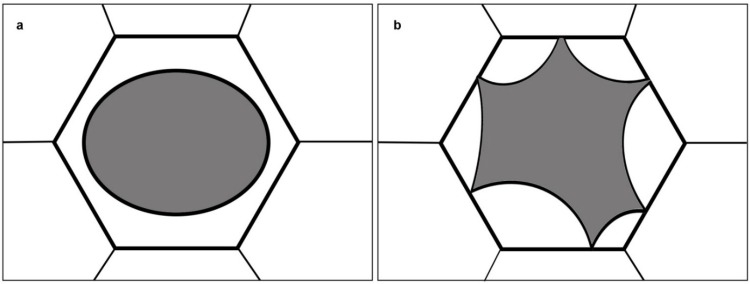
Schematic of the two major plasmolysis forms; (**a**) convex plasmolysis; (**b**) concave plasmolysis.

The process of plasmolysis is probably still known to many from their student days. In hyperosmotic solutions such as sucrose, mannitol or sorbitol, water is extruded from the vacuole causing a loss of turgor pressure. If this state persists, the protoplast retracts further, causing the detachment of the plasma membrane from the rigid cell wall. Two major types of plasmolysis are known, depending on: The cell type, the viscosity of the cytoplasm, and the osmoticum used [1]. In convex plasmolysis, the protoplast is rounded up exhibiting symmetrical convex ends (Figure 1a). In concave plasmolysis, the plasma membrane separates from the cell wall by the formation of several concave pockets (Figure 1b). Plasmolysis is reversible and the addition of hypotonic solutions or plain water will lead to the re-expansion of the protoplast and the reinstatement of the original turgor pressure [1]. The central vacuole is the major compartment of osmotic water flow during plasmolysis but obviously, the abrupt change in protoplast size and shape impacts the subcellular architecture as a whole. In this research, we followed the organization of plant cytoskeletal elements namely: Cortical microtubules and actin microfilaments, during a plasmolytic cycle and documented the entire process in living cells.

Aside from the central vacuole, a rigid cell wall is required for plasmolysis. This structure forms a solid shell encasing the osmo-sensitive and membrane-covered protoplast. Consequently, plasmolysis occurs in walled cell types ranging from bacteria, to fungi and finally plants [1,2,3,4]. When large molecules with a size above the cell wall exclusion limit are used as osmotica (e.g., polyethylene glycols with a MW above 20 kDa) [5], then hyperosmolarity induces the collapse of the entire cell wall—plasma membrane continuum in a phenomenon known as cytorrhysis [6].

In basic biology courses, plasmolysis is used to demonstrate plant cell turgor and its relation to the mechanical rigidity of plant organs. During plasmolysis, the plasma membrane is separated from the cell wall, and this process is easily demonstrated. Specific chemicals like potassium salts lead to swelling of the cytoplasm thereby allowing a distinction between the tonoplast and the plasma membrane in so called cap plasmolysis [7,8]. Furthermore, plasmolysis is an active process characteristic of viable cells; therefore, it is used to test the cellular viability against treatments including heavy metals and other stress factors [9,10,11]. The connection of the plasma membrane and the cell wall is still widely discussed subject [4,12,13,14,15,16] and plasmolysis is essentially used to analyze the space and link between these two structures.

The term plasmolysis was defined by de Vries [17] upon the invention of a method to determine a plant’s turgor pressure using hypertonic solutions. Later, Hecht [18] intensively studied plasmolysed onion epidermal cells. He observed a network-like structure and fine strands (Hechtian strands) in the periplasmic space (the space between the cell wall and the retracted protoplast) that link the protoplast to the inner side of the cell wall of plasmolysed cells. Plasmodesmata have been discussed as candidates for Hechtian attachment sites; these minute channels between adjacent cells have been magnificently observed by transmission electron microscopy in plasmolysed plant tissue [19,20]. However, Hechtian strands can also be formed in the periplasmic space despite cell walls lacking plasmodesmata (e.g., trichomes or outer epidermis walls), suggesting additional structural connections like RGD-containing peptides [13,14], arabinogalactan proteins [21] or growing cellulose microfibrils [22]. A comprehensive review on the process of plasmolysis is given by Oparka [1]. Since then, cytoskeletal elements have been analyzed during a plasmolytic cycle in various plant species and cell types, [23,24,25] but mainly in fixed plant material which was used to visualize microtubules and/or actin microfilaments by means of immunolocalization. In this research, the plasmolytic fate of cortical microtubules and actin microfilaments was followed in epidermal hypocotyl cells of appropriate *Arabidopsis* lines stably transformed with GFP-tagged cytoskeletal markers allowing the documentation of the whole process *in vivo*.

## 2. Results and Discussion

### 2.1. A Plasmolytic Cycle

During a plasmolytic cycle, the semipermeable membranes, plasma membrane and tonoplast, were forced to adjust to the loss of water from the vacuole in hypertonic solutions (plasmolysis), or to the water uptake until full turgor is reinstated (deplasmolysis). Plasmolysis started immediately after contact with the plasmolytic solution and in *Arabidopsis* hypocotyl cells, it was complete after 30 min following exposure to 0.8 M mannitol solution. Cells survived in the plasmolysed state for longer than 24 h, depending on the experimental design and cell type used, [26,27,28] while in exceptional cases they recovered after prolonged exposure to hyperosmoticum reinstating turgor and cortical microtubule organization, suggesting the function of volume regulatory increase mechanisms [26] and references therein, [29]. Fine Hechtian strands and a network like structure (Hechtian reticulum) provided the contact of the plasma membrane to the cell wall during plasmolysis, while preserving the plasma membrane surplus resulting from protoplast reduction [22,26,30,31]. The process of plasmolysis was easily observed with good bright field optics. However, the investigation of subcellular changes in living cells required the use of GFP-tagged *Arabidopsis* lines, as is recognized in the present study using microtubule associated protein 4 (MAP4; [32]) and tubulin alpha 6 (TUA6; [33]) lines for labeling microtubules, and a GFP-tagged actin binding domain of fimbrin 1 (ABD; [34]) for visualization of actin microfilaments in living cells.

### 2.2. Microtubules

**Figure 2:**
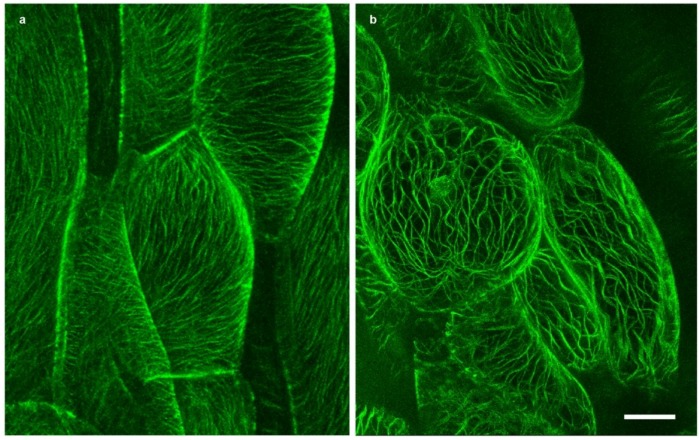
Green fluorescent protein (GFP)-tagged microtubules (GFP-MAP4) in *Arabidopsis* hypocotyl cells; (**a**) Interphase cells before plasmolysis; (**b**) Bundles and wavy microtubules in plasmolysed cells, treatment with 0.8 M mannitol for 30 min; bar: 10 µm.

**Figure 3:**
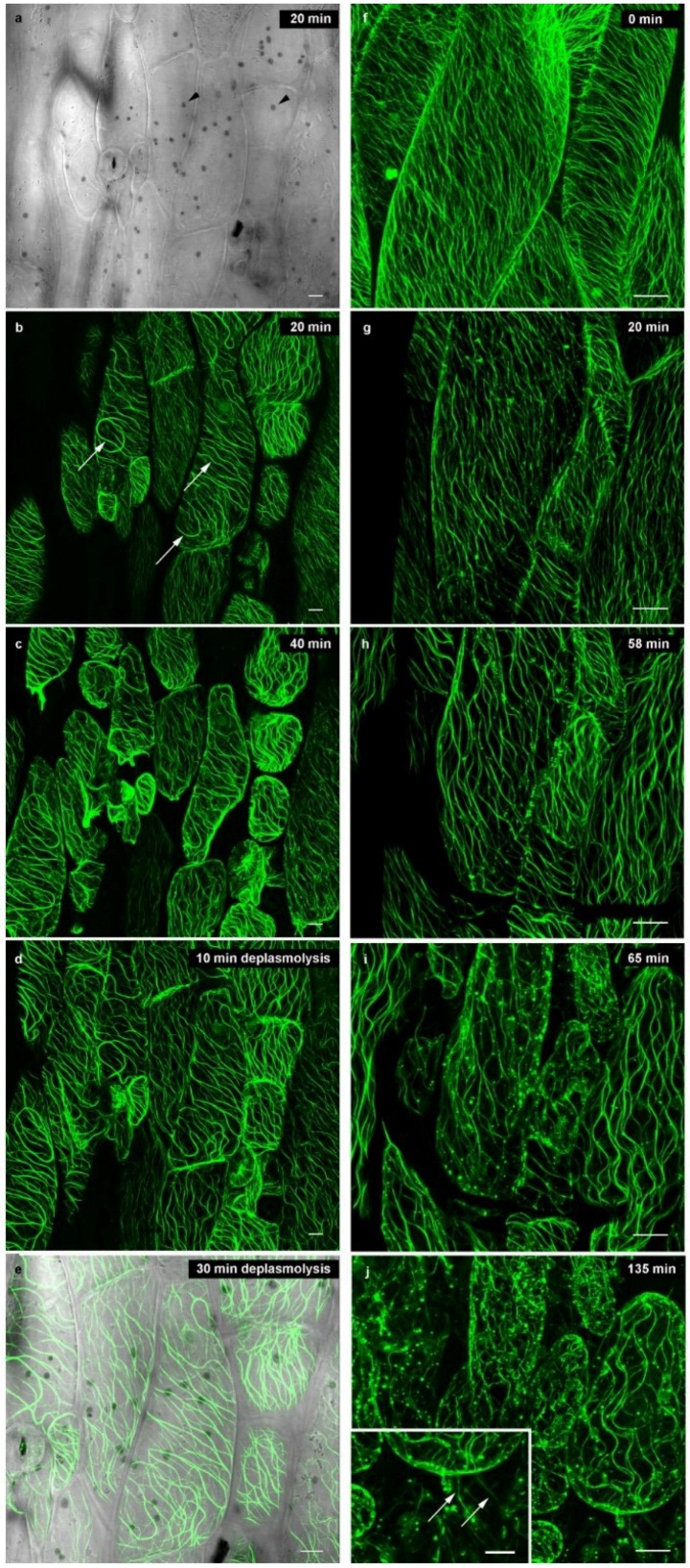
Cortical microtubules (GFP-MAP4) in *Arabidopsis* hypocotyl cells during plasmolysis (**a**–**c**; **f**–**j**) and deplasmolysis (**d**,**e**); plasmolysis/deplasmolysis times marked on the micrographs. (**a**) Bright field image showing cell walls, occasional stomata and chloroplasts (arrowheads); (**b**) Bundling of microtubules (arrows) at the onset of plasmolysis in 0.8 M mannitol; (**c**) After 40 min, cortical microtubules are showing waves and bundles; they remain aligned within the cortical cytoplasm; (**d**) Bundles of microtubules persist in deplasmolysis when the protoplasts become realigned at the cell wall; (**e**) Overlay of fluorescence and bright field image at the end of the plasmolytic cycle; chloroplasts appear as dark grey dots; (**f**–**j**) Higher magnification of plasmolysing hypocotyl cells; inlay in (**j**) shows microtubules in Hechtian strands (arrows). Bar: 10 µm.

In interphase cells, plasmolysis (which is the disruption of the cell wall—plasma membrane—cortical cytoskeleton continuum) is expected to exert the strongest impact on cortical microtubules since they are closely linked to the plasma membrane, exerting a role in oriented cellulose microfibril deposition [15,16,28,35,36,37]. In fully turgid *Arabidopsis* hypocotyl cells, we observed the biased organization of cortical microtubules arranged in parallel order, and in oblique to transverse orientations in the cortical cytoplasm (Figure 2a and Figure 3f). At the onset of plasmolysis, cortical microtubules became wavy in order to accommodate the decreased shape of the protoplast (Figure 2b). Concurrently, the microtubules come together to form bundles (Figure 3). In the bundled state, cortical microtubules were preserved for up to 24 h while still in the plasmolytic medium. Interestingly, the cellulase KORRIGAN (KOR) showed similar microtubule arrays [38] but a role of microtubules in the transport of cellulose synthase (CESA) proteins from their origin in the Golgi towards the plasma membrane, e.g., by microtubule-associated CESA compartments (MASCs) or small CESA compartments (SmaCCs) is still under discussion (for a review, see [39]). Cortical microtubules labeled with GFP-MAP4 and GFP-TUA6 exhibit the same behavior under plasmolytic conditions but the background in GFP-TUA6 cells was higher (Supplemental Figure S1). Therefore, only data for GFP-MAP4 are submitted hereafter. Microtubules or microtubule bundles were also present in Hechtian strands (Figure 3j). In deplasmolysis, microtubules remained organized in thick bundles, but they gradually separated from each other during the course of the protoplast swelling to its original size (Supplemental Movie S1). In some samples, fluorescently labeled spots are observed (Figure 3h–j); these are located along microtubule bundles. It took up to 24 h until fine cortical microtubules were reinstated but even then, some thick tubular structures—probably corresponding to residual microtubule bundles—persisted (data not shown).

### 2.3. Actin Microfilaments

**Figure 4:**
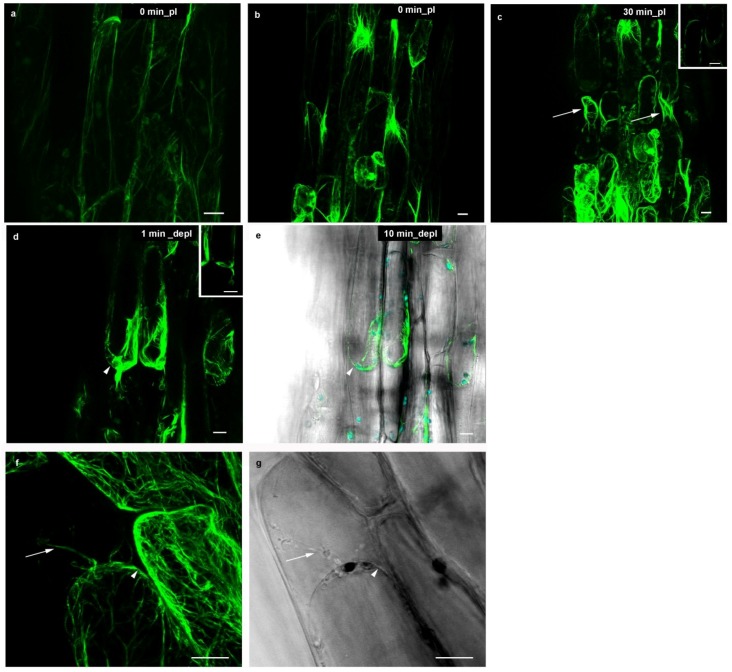
Actin microfilaments (GFP-ABD) in *Arabidopsis* hypocotyl cells during a plasmolytic cycle; plasmolysis/deplasmolysis times marked on the micrographs. (**a**,**b**) Before plasmolysis, actin microfilaments were located in the cortical cytoplasm (**a**) and radiated from the nucleus (**b**); (**c**) In the plasmolysed state, actin microfilaments conserved this structure, but also lined the detached plasmalemma regions (arrows); (**d**) The protoplast rounded up at the onset of deplasmolysis, incorporating Hechtian strands (arrowheads) and (**e**) a “stabilizing layer” of bundled actin microfilaments followed the expanding protoplast; (**f**,**g**) High magnification of Hechtian strands (arrows) with actin microfilaments (**f**) and the corresponding bright field image (**g**), 30 min of plasmolysis in 0.8 M manntiol; arrowheads indicate the plasma membrane. Inserts (**c**,**d**): Single focal planes to show the cortical array. Bar: 10 µm.

Filamentous actin, as visualized by GFP-ABD in *Arabidopsis* hypocotyl cells, presented a fine network (Figure 4a). It consisted of radiating microfilaments and microfilament bundles which extended from the nuclear envelope towards the cortical parts of the cell (Figure 4b). Endoplasmic actin was present in transvacuolar cytoplasmic strands that traversed the vacuole thereby connecting the nuclear cytoplasm to the thin cortical cytoplasmic layer immediately below the plasma membrane. Since the subcortical cytoplasm was highly dynamic and organelles therein are constantly streaming in interphase cells, actin microfilaments needed to adjust accordingly. Therefore, it is easily understandable that actin microfilaments were able to reorganize rapidly in order to accommodate the shrinking protoplast during plasmolysis. The actin filaments radiating from the nuclear surface were still visible in plasmolysed cells (Figure 4b,c), while cortical actin lined the plasma membrane (arrowheads; Figure 4c,d, inserts) and stretched out towards the cell wall in Hechtian strands (Figure 4f,g, arrows). The actin filaments did not become wavy as observed for microtubules. The plant cytoplasm contained a pool of globular actin in order to constantly assemble filaments; these in turn can rapidly disassemble if F-actin remodeling is necessary. Actin microfilament reorganization after hyperosmotic treatment was linked to Ca^2+^ signaling and was associated with membrane mechanical integrity ([26] and references therein). However, it remains to be clarified if this Ca^2+^ mobilization triggered actin filament formation directly by the increased polymerization of globular actin [40], or indirectly through a Ca^2+^ signaling cascade [41].

At the onset of deplasmolysis, filamentous actin was clearly visible at the sites of big cytoplasmic aggregations: Around the nucleus and lining of the concave membrane-wall detachments. A weak fluorescent signal of GFP-ABD protein was observed along the plasma membrane of the expanding protoplast, even when there was only a very thin cortical layer. Hechtian strands became incorporated into the expanding protoplast. The cytoarchitecture at the start of deplasmolysis was preserved throughout the process (Figure 4d,e/Supplemental Movie S2) until the protoplast was reconnected with the cell wall. Shortly after the plasmolytic cycle was completed, actin microfilaments in *Arabidopsis* hypocotyl cells were organized similarly to non-plasmolyzed control cells. Consequently, most filamentous actin was found to be present at sites of high cytoplasmic density as well as in the thin cortical layer below the plasma membrane. Organelle movement denoting cytoplasmic streaming was maintained throughout the whole plasmolytic cycle (Supplemental Movie S3). In leaf cells of *Chlorophytum comosum*, the behavior of cortical actin filaments during plasmolysis has been described in terms of plasma membrane integrity and protoplast volume regulation [26]. In this study, actin microfilaments were shown to line the plasma membrane at areas of intense mechanical strain as in the case of concave plasmolysis. The authors report that many actin filament bundles formed a network, lining the areas of detached plasma membrane during concave plasmolysis. In addition, these fibers were compared to stress fibers found in animal cells. It is suggested that the formation of numerous cortical, subcortical and endoplasmic actin filaments was necessary to regulate shape and volume in plasmolysis (see also [42,43]). Furthermore, Komis and co-workers [26] describe the disappearance of thin cortical actin filaments during plasmolysis. This, however, could be attributed to the fact that cells were fixed and most of the actin in the cortical layer was lost. The GFP-tagged actin *Arabidopsis* line in the present study allowed for the use of living material. A faint fluorescence signal was observed along the protoplast, even at sites that contain hardly any cytoplasm.

### 2.4. A Broader View

Plasmolysis is not only used in laboratory experiments, it has been reported to occur naturally due to extracellular water withdrawal in freezing conditions [44]. A lot of current knowledge on the water balance of plants is based on extensive studies by Stadelmann [5] and his co-workers. In this study, although the focus was maintained on the cytoskeleton, keeping in consideration that it is part of a complex ER-cytoskeleton-plasma membrane-cell wall continuum, and plays an essential role in signaling and mechanosensing [21,45,46].

## 3. Experimental Section

Hypocotyl cells of *Arabidopsis thaliana* plants were used in this study. The plants were grown on ½ MS medium [47] under sterile culture conditions for five days. Apart from wild-type plants (Col 0), cytoskeletal elements were followed in green fluorescent protein (GFP)-tagged Arabidopsis lines. In order to visualize microtubules, *Arabidopsis* lines expressing a microtubule associated protein coupled to GFP (GFP-MAP4; [32]) were utilized; seeds were generously gifted by Professor Jozef Šamaj as well as an alpha tubulin encoding line (GFP-TUA6; [33]); kind gift from Dr. Sidney Shaw. Actin microfilaments were detected in *Arabidopsis* plants expressing a GFP-tagged actin binding domain of fimbrin 1 (GFP-ABD; [34]); another kind gift from Professor Jozef Šamaj.

Seedlings were secured between a microscope slide and a coverslip spaced by Parafilm stripes, and sealed using liquid petroleum jelly. The immobilization in the application of petroleum jelly prevented dislocation of the plantlets during liquid exchange and allowed for the observation of the same cells during a whole plasmolytic cycle. To induce plasmolysis, a 0.8 M mannitol solution was applied on one side of the coverslip and carefully removed from the opposite side using filter paper. Plasmolysis was completed after 30 to 40 min. Deplasmolysis was initiated by perfusing a 0.4 M mannitol solution for 15 min followed by the perfusion of distilled water.

Both plasmolysis and deplasmolysis, were observed under a confocal laser scanning microscope (Leica CTR SP5 linked to a DM6000CS stand) using a 63× water immersion lens. Z-stacks were taken at specific time frames (e.g., onset of plasmolysis, after 10 min, 30 min, 60 min, 12 h, and finally after 24 h). Time lapse video clips were also produced to follow the dynamic processes. For the videos shown as supplemental data, single images were taken at 2 min time intervals. The single image series were exported as .avi files by the Leica LASAF software at 12 fps. Editing was done with Adobe Premiere Pro version CS4 and CS6 (Adobe, San Jose, CA, USA).

## 4. Conclusions

Although plasmolysis is used in many cell biology experiments and student courses, the process itself and the incurred cytoarchitectural rearrangements remain to be fully understood. In this research paper, we describe cortical microtubule and actin microfilament organization during a plasmolytic cycle. Both structures are forced to adjust accordingly in the diminishing/expanding protoplast driven by vacuolar water efflux/influx. Further functional studies using stabilizing or disrupting agents on the cytoskeleton will allow for a more in-depth view on the role of cytoskeletal elements in plasmolysis.

## Supplementary Files

Supplementary File 1
