# Supplementary material for: Plasmolysis: Loss of Turgor and Beyond
Source: Plants (Basel). 2014 Nov 26;3(4):583–93. doi: 10.3390/plants3040583 (PMC4844282; doi:10.3390/plants3040583)
Supplement: Supplementary File 1 [file plants-03-00583-s001.zip › plants-03-00583-Supplement/Supplemental Material & Figure S1.pdf]

Supplementary Material

Figure S1. *Arabidopsis* wild type (Col0) has been used as a control (**a**–**c**). It has been treated the same way as the transgenic reporter lines. The Supplemental Figure plate S1 is showing Col0, GFP-MAP4, GFP-TUA6 and GFP-ABD in the non-plasmolysed state (**a**,**d**,**g**,**h**) as well as in the plasmolysed state after 30 min in 0.8 M mannitol (fluorescent images **b**,**e**,**h**,**k** and bright field **c**,**f**,**i**,**l**). Bar: 10 µm.

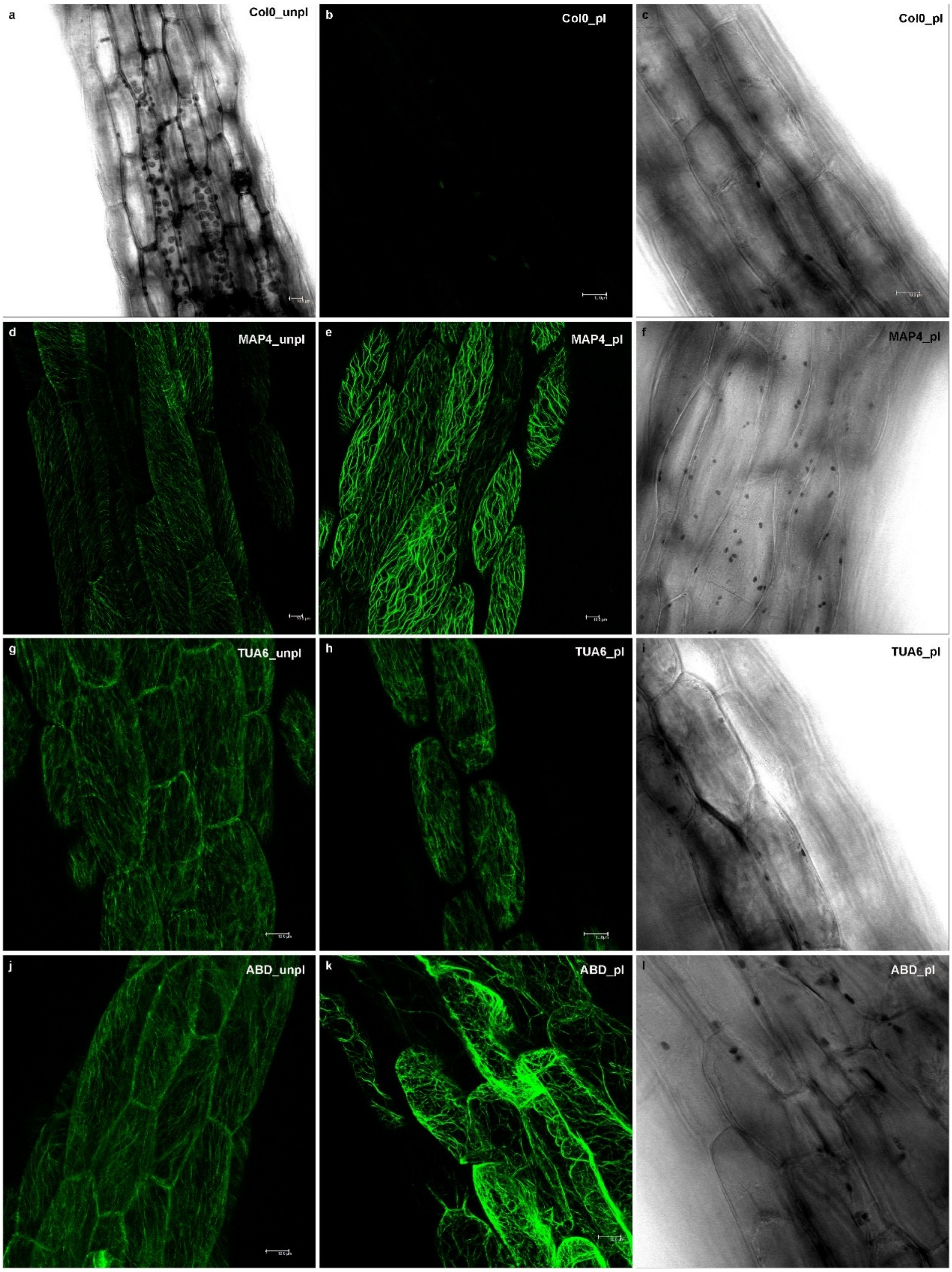

Movies S1–3

Movie S1

Distribution and motility of GFP-MAP4 in plasmolysis and deplasmolysis (0.8 M mannitol), overlay of fluorescent and bright field images. Time lapse movie of the *Arabidopsis* hypocotyl cells shown in Figure 3. Movie acquired over 20 min, images acquired at 2 min intervals.

Movie S2

Fluorescent images of a GFP-ABD line of *Arabidopsis* showing endoplasmic actin and cortical actin in hypocotyl cells. Actin is visible in the cortex of the protoplast in plasmolysis and also in deplasmolysis. Hechtian strands are incorporated when the protoplast expands in deplasmolysis. Movie acquired before plasmolysis, after 30 min of plasmolysis and over 20 min during deplasmolysis, all images acquired at 2 min intervals.

Movie S3

Evidence for dynamics of cytoplasmic organelles in bright field mode during plasmolysis and expansion of the cells in deplasmolysis. Images acquired every min, over a period of 20 min.

© 2014 by the authors; licensee MDPI, Basel, Switzerland. This article is an open access article distributed under the terms and conditions of the Creative Commons Attribution license (http://creativecommons.org/licenses/by/4.0/).
